# EFEMP2 Is Associated with Shelterin-Related DNA Damage Repair, Immune Microenvironment Features, and Prognosis in Glioblastoma

**DOI:** 10.3390/biomedicines14081785

**Published:** 2026-08-07

**Authors:** Jiaxiang Wang, Chunbo Liu, Fushu Luo, Yongye Zhu, Zheng Chen, Yutao Zhang, Changwu Wu, Qing Liu, Jun Tan

**Affiliations:** 1 Department of Neurosurgery, Xiangya Hospital, Central South University, Changsha 410008, China; xyyydr_wang@csu.edu.cn (J.W.);; 2 National Clinical Research Center for Geriatric Disease, Xiangya Hospital, Central South University, Changsha 410008, China; 3 Clinical Research Center for Skull Base Surgery and Neuro-Oncology in Hunan Province, Changsha 410008, China

**Keywords:** glioblastoma, shelterin complex, EFEMP2, DNA damage repair, tumor microenvironment

## Abstract

**Background:** Glioblastoma (GBM) carries a median overall survival below 15 months despite aggressive multimodal therapy. Treatment resistance reflects the interplay of genomic instability, dysregulated DNA damage repair (DDR), and an immunosuppressive tumor microenvironment (TME). The shelterin complex maintains telomere integrity, yet its broader role in coordinating DDR, TME remodeling, and clinical outcomes in GBM remains unclear. **Methods:** We integrated transcriptomic and clinical data from four public cohorts (TCGA, CGGA1, CGGA2, and REMBRANDT, *n* = 583) and an institutional cohort (CSUXY, *n* = 65). A composite shelterin score was computed by ssGSEA and correlated with DDR activity, immune infiltration, and checkpoint expression. Ten machine-learning algorithms generated 101 candidate model configurations; the final shelterin-related signature (SRS) was trained in TCGA and evaluated in external public cohorts and the institutional cohort. EFEMP2, the top-weighted gene in the SRS, was further examined using public transcriptomic datasets, an exploratory anti-PD-1 cohort, immunohistochemistry, and in vitro assays. **Results:** Higher shelterin scores were associated with enhanced DDR pathway activity, greater immune and stromal infiltration, and elevated checkpoint expression. The SRS achieved moderate discrimination, with a mean C-index of 0.60, and stratified patients into prognostically distinct risk groups across cohorts. EFEMP2 was consistently associated with inferior overall survival. High EFEMP2 correlated with an immunosuppressive TME enriched for MDSCs and exhausted T cells alongside reduced enrichment of several DDR pathways. Silencing EFEMP2 suppressed proliferation, migration, and invasion while inducing DNA double-strand break markers. In the small anti-PD-1 cohort, high EFEMP2 showed non-significant trends toward longer OS and PFS, which should be interpreted as hypothesis-generating. **Conclusion:** This study provides a biologically interpretable prognostic framework that was externally evaluated across multiple independent cohorts and links shelterin-related biology to GBM outcomes. EFEMP2 may connect genomic stress and immune suppression, but its mechanistic and immunotherapy-predictive roles require further validation.

## 1. Introduction

Glioblastoma (GBM) is the most lethal primary brain malignancy of adulthood, defined by rapid progression, pervasive intratumoral heterogeneity, and a median overall survival that rarely exceeds 15 months under current standard-of-care therapy [1,2]. Beyond diffuse infiltrative growth, the biological aggressiveness of GBM reflects a dual capacity: the ability to withstand genotoxic stress through efficient DNA damage repair (DDR), and the ability to co-opt and reshape the tumor microenvironment (TME) toward immunosuppression [3,4,5]. Because these tumor-intrinsic and microenvironmental programs interact to drive therapeutic resistance and recurrence, identifying shared molecular regulators of DDR and TME reprogramming could inform new therapeutic approaches.

Telomeres, repetitive nucleoprotein caps at chromosome termini, are indispensable for chromosomal stability, and their functional integrity depends on the shelterin complex, a hexameric assembly of TERF1, TERF2, TERF2IP, TINF2, TPP1, and POT1 [6,7]. By capping chromosome ends, the shelterin complex prevents their recognition as DNA lesions and coordinates telomere-length homeostasis with DNA repair processes. Loss of shelterin function triggers telomere deprotection, replication stress, chromosomal end-to-end fusions, and activation of ATM/ATR-dependent damage signaling, all of which fuel chromosomal instability and can accelerate tumor evolution [8,9,10]. In glioma, telomere biology is implicated in disease pathogenesis through telomerase reactivation and alternative lengthening of telomeres; however, whether and how shelterin-related transcriptional programs relate to DDR capacity, TME composition, and patient survival in GBM remain open questions [11,12,13].

Genome instability ranks among the defining hallmarks of GBM and is tightly coupled to disease progression, recurrence, and treatment failure [14,15]. Recent studies have shown that DDR defects can alter antitumor immunity by modulating inflammatory signaling, neoantigen generation, and immune checkpoint expression [16,17,18]. The GBM microenvironment is marked by pronounced immune dysregulation, stromal remodeling, hypoxia, and extensive crosstalk between tumor cells and infiltrating immune populations [19]. Although GBM has traditionally been classified as an immunologically “cold” tumor, recent single-cell and spatial transcriptomic studies have revealed substantial heterogeneity in immune infiltration patterns, with immune cell recruitment frequently coexisting with T-cell exhaustion, myeloid-mediated suppression, and upregulation of immune checkpoint ligands rather than translating into effective antitumor responses [20,21,22,23]. It remains unexplored whether shelterin-related molecular features contribute to this immunosuppressive phenotype or, conversely, define tumor subsets more amenable to immune checkpoint blockade.

Machine learning offers a powerful framework for extracting robust prognostic signatures from high-dimensional transcriptomic data. Compared with univariate biomarker approaches, integrative multi-algorithm strategies better accommodate gene-gene collinearity, improve model stability, and enhance external generalizability [24,25]. Despite these advantages, many published GBM prognostic models suffer from limited cross-cohort reproducibility and shallow biological interpretability, especially with respect to DDR and immune contexture. A prognostic framework that is both computationally robust and biologically transparent therefore remains an unmet need.

Here, we undertook a systematic dissection of the clinical and biological relevance of shelterin-related features in GBM. We first assessed the prognostic value of the six canonical shelterin genes and a composite shelterin score, and then mapped their associations with DDR activity, TME composition, immune checkpoint expression, and immune cell infiltration. To sharpen risk stratification, we benchmarked 101 prognostic models generated by combining ten machine learning algorithms and selected the optimal shelterin-related signature (SRS). EFEMP2, the gene contributing the largest coefficient to the final model, was prioritized for in-depth characterization of its associations with the immune microenvironment, DDR pathways, and exploratory anti-PD-1 outcomes, and was further evaluated by immunohistochemistry and in vitro functional assays. By bridging telomere protection biology, DDR transcriptional programs, immune microenvironment profiling, and machine learning-based risk modeling, this study aimed to define a biologically interpretable framework for GBM progression and identifies EFEMP2 as a molecular node of potential translational relevance, while explicitly recognizing the need for further mechanistic validation.

## 2. Materials and Methods

### 2.1. Data Acquisition and Study Cohorts

Sixty-five fresh GBM tissue specimens were collected from the Department of Neurosurgery at our institution following written informed consent. The study was approved by the Ethics Committee of Xiangya Hospital, Central South University (Approval No. 202401003) and was performed in accordance with the Declaration of Helsinki. Clinical and follow-up data were compiled to establish institutional validation cohort CSUXY. Publicly available GBM datasets (TCGA, CGGA1, CGGA2, and REMBRANDT) were obtained from GlioVis (http://gliovis.bioinfo.cnio.es/, accessed on 15 June 2026) and the Gene Expression Omnibus (GEO; https://www.ncbi.nlm.nih.gov/geo/, accessed on 15 June 2026) [26]. After quality control, bulk RNA-seq data with complete clinical and prognostic annotation were available for 141 (TCGA), 199 (CGGA1), 134 (CGGA2), and 109 (REMBRANDT) cases. Clinical and follow-up data were compiled to establish the institutional CSUXY validation cohort. This cohort was derived from 65 GBM cases used in the present GBM analysis. Available CSUXY annotations included clinical features, OS status and follow-up time, histology, radiotherapy, chemotherapy, and immunohistochemical markers. The CSUXY cohort was used for institutional survival-based validation and tissue-level EFEMP2 immunohistochemical support; it was not used for model training or for the full set of transcriptomic association analyses. To evaluate exploratory associations with immune checkpoint blockade (ICB) outcome, we incorporated an independent anti-PD-1 immunotherapy cohort from a published study [27], which included 37 recurrent GBM patients treated with neoadjuvant and adjuvant anti-PD-1 monotherapy, with complete transcriptomic data and OS/PFS clinical endpoints.

### 2.2. Definition of the Shelterin Gene Set and Calculation of the Shelterin Score

The shelterin complex was represented by its six canonical core subunits: TERF1, TERF2, TERF2IP, TINF2, TPP1, and POT1. For each sample, a composite shelterin score reflecting overall complex activity was derived from the normalized expression of these six genes using ssGSEA, implemented in the GSVA R package (v1.46.0) [28]. ssGSEA was chosen because it accommodates variation in gene-level magnitudes and provides a normalized enrichment metric suitable for cross-cohort comparison. This score was carried forward into all downstream correlation, survival, and stratification analyses. Samples were dichotomized into high- and low-score groups based on the median shelterin score. Differentially expressed genes between the two groups were identified using the “limma” R package and designated as shelterin-related genes (SRGs). The threshold for differential expression analysis was set to log2|fold change| > 0.5 and *p* < 0.05. Subsequently, Kyoto Encyclopedia of Genes and Genomes (KEGG) pathway analysis and Gene Ontology (GO) biological processes enrichment analysis were performed on SRGs using the R package “clusterProfiler” [29]. SRGs were shown in Additional file: Table S1.

### 2.3. Survival and Pathway Analyses

For each shelterin gene and the composite shelterin score, patients were dichotomized into high- and low-expression groups at the optimal cutoff determined by the surv_cutpoint function (survminer R package, v0.4.9). OS was compared between groups by Kaplan–Meier curves, with statistical significance assessed by the two-sided log-rank test. Hazard ratios (HRs) with 95% confidence intervals (CIs) were estimated using univariable Cox proportional hazards models for the corresponding survival comparisons. Because data-driven optimal cutoffs can introduce optimism and may not transfer directly to clinical practice, these cutoffs were used for retrospective exploratory stratification and require future validation in prospectively defined cohorts. Hallmark pathway activity was quantified by ssGSEA across the 50 MSigDB gene sets [30], and differential activation between shelterin-score groups was evaluated with the Wilcoxon test. DDR pathway activity was quantified by gene set variation analysis (GSVA) and correlated with the shelterin score or SRS using Spearman rank correlation.

### 2.4. Tumor Microenvironment and Immune Infiltration Analysis

TME composition was assessed using the ESTIMATE and CIBERSORT algorithms. ESTIMATE (estimate R package) was applied to infer stromal, immune, and overall ESTIMATE scores together with tumor purity from bulk transcriptomic data [31]. Relative abundances of 22 immune cell subsets were estimated by CIBERSORT with the LM22 reference matrix and 1000 permutations [32]. Twenty-eight additional immune cell subtypes were quantified by ssGSEA. Expression of immune checkpoint genes was correlated with the shelterin score, SRS, and EFEMP2 expression by Spearman analysis.

### 2.5. Construction of the Machine Learning-Based Shelterin-Related Prognostic Signature (SRS)

A machine learning framework integrating ten algorithms was implemented [33,34]: random survival forest (RSF), elastic net (Enet), Lasso, Ridge regression, stepwise Cox regression (StepCox), CoxBoost, partial least squares regression for Cox (plsRcox), supervised principal components (SuperPC), generalized boosted regression modeling (GBM), and survival support vector machine (survival-SVM). Pairwise and serial combinations of these algorithms yielded 101 candidate model configurations. Candidate genes for modeling comprised those significantly associated with OS by univariate Cox regression (*p* < 0.05) in the TCGA-GBM training cohort, which were shown in Additional file: Table S2. Model training, feature selection, and hyperparameter tuning were conducted exclusively in the TCGA-GBM training cohort. The 101 candidate configurations were then applied to the independent REMBRANDT, CGGA1, CGGA2, and CSUXY cohorts, and their predictive performance was quantified using Harrell’s C-index. Regression coefficients for retained genes were obtained from multivariable Cox proportional hazards models, and an individualized SRS risk score was computed as SRS = Σ(βi × Expi), where βi is the Cox coefficient and Expi the normalized expression of gene i. Patients in each cohort were then stratified into high- and low-risk groups at the optimal cutoff of the SRS risk score (surv_cutpoint), with OS differences evaluated by log-rank test and HR with 95% CI estimated by Cox regression.

### 2.6. In-Depth Analyses of EFEMP2

EFEMP2, the gene with the largest coefficient in the final SRS model, was prioritized for detailed characterization. Patients were stratified at the optimal expression cutoff, and survival was evaluated by Kaplan–Meier analysis with HR and 95% CI estimated by Cox regression. Biological associations of EFEMP2 were assessed through ssGSEA, ESTIMATE, CIBERSORT, and immune checkpoint correlation analyses. In the anti-PD-1 cohort, OS and PFS were compared between high- and low-EFEMP2 groups as an exploratory analysis, because the cohort was small and both endpoints were considered hypothesis-generating unless statistically supported.

### 2.7. Cell Lines and Culture Conditions

The human glioma cell line U251 and the human osteosarcoma cell line U2OS were obtained from the Xiangya Cell Repository. U251 cells were maintained in Dulbecco’s modified Eagle’s medium (DMEM; Corning, NY, USA) supplemented with 10% fetal bovine serum (FBS; Auckland, New Zealand) at 37 °C under 5% CO_2_. U2OS cells were grown in McCoy’s 5A medium (Abiowell, Changsha, China) under identical conditions. Small interfering RNAs (siRNAs) targeting EFEMP2 (si-EFEMP2) and a non-targeting negative control (si-NC) were synthesized by Sangon Biotech (Shanghai, China). Three candidate siRNAs were screened for knockdown efficiency; the two with superior silencing were selected and designated siEFEMP2-1 and siEFEMP2-2. U251 was used as the GBM-lineage cellular model. U2OS, a DDR-proficient model widely used for DNA damage studies, was included only as a supportive system to test whether EFEMP2 depletion was accompanied by DNA damage phenotypes in a DDR-competent context. It was not intended to substitute for primary GBM validation.

### 2.8. Quantitative Reverse Transcription-Polymerase Chain Reaction (RT-qPCR)

Total RNA was isolated from cells cultured in 6-well plates using 1 mL of AG RNAex Pro Reagent (Accurate Biology, Changsha, China). Phase separation was performed with chloroform, followed by RNA precipitation with isopropanol and washing with 75% ethanol. Purified RNA was dissolved in RNase-free water, and concentration and purity were determined spectrophotometrically. cDNA was synthesized from equal amounts of total RNA using a reverse transcription kit (Accurate Biology, Changsha, China). Quantitative PCR was performed with a SYBR Green-based master mix on a real-time PCR system under the manufacturer’s recommended cycling conditions. Relative expression was calculated by the 2^−^^ΔΔCt^ method with GAPDH as the internal reference. All reactions were run in triplicate, and experiments were independently repeated at least three times.

### 2.9. Cell Proliferation Assay

Cells were transfected with RNATransMate reagent (Sangon Biotech, Shanghai, China) according to the manufacturer’s instructions. At 24 h post-transfection, cells were seeded into 96-well plates at 2 × 10^3^ cells per well. Cell viability was measured at 0, 1, 2, and 3 days by adding 10 μL of Cell Counting Kit-8 (CCK-8; Meilunbio, Dalian, China) reagent per well and incubating for 3 h at 37 °C, followed by absorbance measurement at 450 nm.

### 2.10. Cell Invasion and Migration Assays

Migratory capacity was evaluated by wound-healing assay: a linear scratch was made on a confluent monolayer, and closure was monitored over 36 h. Invasive capacity was assessed by Transwell assay: cells in serum-free medium were seeded into the upper chamber over a Matrigel-coated membrane, with 10% FBS-containing medium in the lower chamber serving as chemoattractant. After 24 h, cells that had traversed the membrane were fixed, stained, and counted under a microscope.

### 2.11. Colony Formation Assay

U251 and U2OS cells subjected to EFEMP2 knockdown were seeded at 1 × 10^3^ cells per well in 6-well plates and cultured for 10 days. Colonies were fixed with 4% paraformaldehyde for 30 min, stained with 0.1% crystal violet, and quantified.

### 2.12. Immunohistochemistry (IHC)

Paraffin-embedded glioma tissue sections were deparaffinized, rehydrated, and subjected to antigen retrieval. After blocking endogenous peroxidase, sections were incubated with rabbit polyclonal anti-EFEMP2 (Proteintech, Wuhan, China; 12004-1-AP; 1:100), followed by the appropriate secondary antibody. Immunoreactivity was visualized with a diaminobenzidine detection system and counterstained with hematoxylin. Staining was independently assessed and graded by two blinded pathologists, with discrepancies resolved by consensus.

### 2.13. Western Blotting

Whole-cell lysates were prepared in RIPA buffer (Epizyme Biotech, Shanghai, China) supplemented with protease and phosphatase inhibitors (Abiowell, Changsha, China). Equal amounts of protein were resolved by SDS-PAGE, transferred to PVDF membranes (Millipore, Billerica, MA, USA), blocked with 5% non-fat milk or BSA for 90 min, and probed overnight at 4 °C with primary antibodies against EFEMP2 (Proteintech, 12004-1-AP; 1:2000) and γ-H2AX (ZenBio, Durham, NC, USA, 201082-7G9; 1:1000). After incubation with HRP-conjugated secondary antibodies for 1 h at room temperature, bands were visualized on an ImageQuant 600 system (Cytiva, Marlborough, MA, USA).

### 2.14. Comet Assay

DNA DSB accumulation was assessed by alkaline single-cell gel electrophoresis using a commercial Comet Assay Kit (Beyotime Biotechnology, Shanghai, China). Harvested cells were embedded in low-melting-point agarose on comet slides, lysed, subjected to electrophoresis, and stained with a DNA-binding fluorescent dye. Comet tail formation was visualized by fluorescence microscopy.

### 2.15. Statistical Analysis

Bioinformatic analyses were performed in R (v4.2.2). Survival analyses used the survival and survminer packages. Group comparisons employed Student’s *t*-test, Wilcoxon rank-sum test, chi-square test, or Fisher’s exact test as appropriate. Correlations were assessed by Spearman rank correlation; multiple testing was corrected by the Benjamini–Hochberg method where applicable. HR with 95% CI was estimated using Cox proportional hazards models for Kaplan–Meier comparisons. In vitro data are presented as mean ± SD from at least three independent experiments and were analyzed by unpaired two-tailed *t*-test or one-way ANOVA in GraphPad Prism (v9.0). Two-sided *p* < 0.05 was considered statistically significant (* *p* < 0.05; ** *p* < 0.01; *** *p* < 0.001; **** *p* < 0.0001).

## 3. Results

### 3.1. Prognostic Significance of Shelterin Complex Genes and the Composite Shelterin Score in GBM

**Figure 1:**
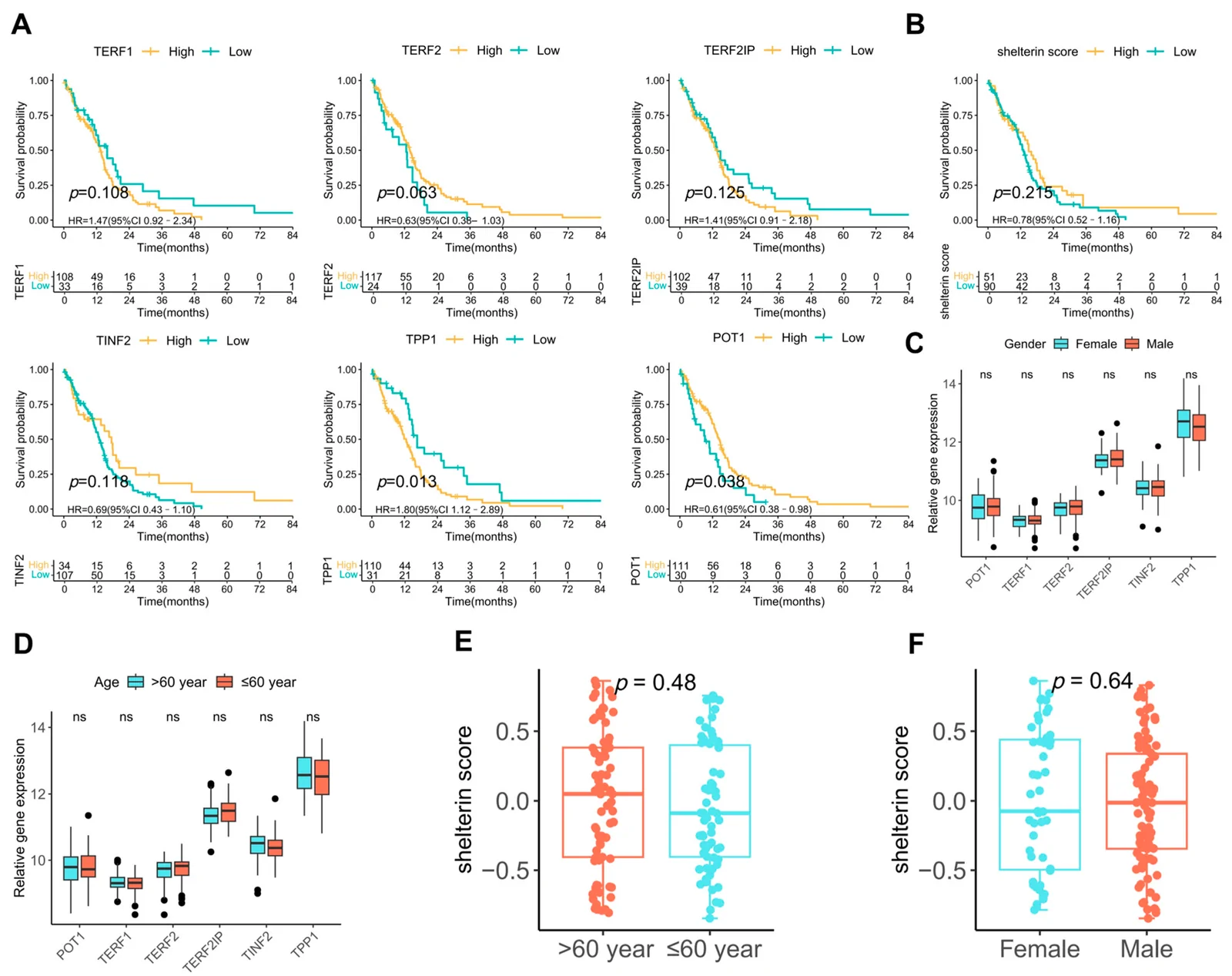
Prognostic significance of shelterin complex genes and the composite shelterin score in GBM. (**A**,**B**) Kaplan–Meier overall survival curves for the six core shelterin genes (TERF1, TERF2, TERF2IP, TINF2, TPP1, and POT1) and the composite shelterin score in the TCGA-GBM cohort, using optimal cutoffs determined by surv_cutpoint. *p* values were determined by log-rank test and HR by Cox regression. (**C**–**F**) Distribution of sex and age between high- and low-expression groups for each shelterin gene and the composite score in the TCGA-GBM cohort, confirming baseline balance (all *p* > 0.05).

Kaplan–Meier survival analysis of the six core shelterin genes and the composite shelterin score revealed significant survival differences between the high- and low-expression groups for TPP1 (HR = 1.80, 95% CI: 1.12–2.89, *p* = 0.013) and POT1 (HR = 0.61, 95% CI: 0.38–0.98, *p* = 0.038), while TERF1 (HR = 1.47, 95% CI: 0.92–2.34, *p* = 0.109), TERF2 (HR = 0.63, 95% CI: 0.38–1.03, *p* = 0.063), TERF2IP (HR = 1.41, 95% CI: 0.91–2.18, *p* = 0.125), TINF2 (HR = 0.69, 95% CI: 0.43–1.10, *p* = 0.118), and the composite shelterin score (HR = 0.78, 95% CI: 0.52–1.16, *p* = 0.215) did not reach significance (Figure 1A,B). Baseline balance assessment confirmed that the distributions of sex and age were comparable between groups for all genes and the shelterin score (all *p* > 0.05) (Figure 1C–F).

### 3.2. Biological Pathways Associated with the Shelterin Score in GBM

ssGSEA of Hallmark gene sets revealed a clear partitioning of pathway activity along the shelterin-score gradient. Low shelterin score samples were enriched for KRAS_SIGNALING_DN, WNT_BETA_CATENIN_SIGNALING, and HEDGEHOG_SIGNALING, suggesting that attenuated shelterin expression may coincide with aberrant activation of oncogenic and cell-cycle-related programs. Conversely, high-shelterin-score samples displayed enrichment of DNA_REPAIR, PI3K_AKT_MTOR_SIGNALING, HYPOXIA, INFLAMMATORY_RESPONSE, and P53_PATHWAY, linking elevated shelterin expression to genomic maintenance, cell survival, inflammatory activation, and stress response.

**Figure 2:**
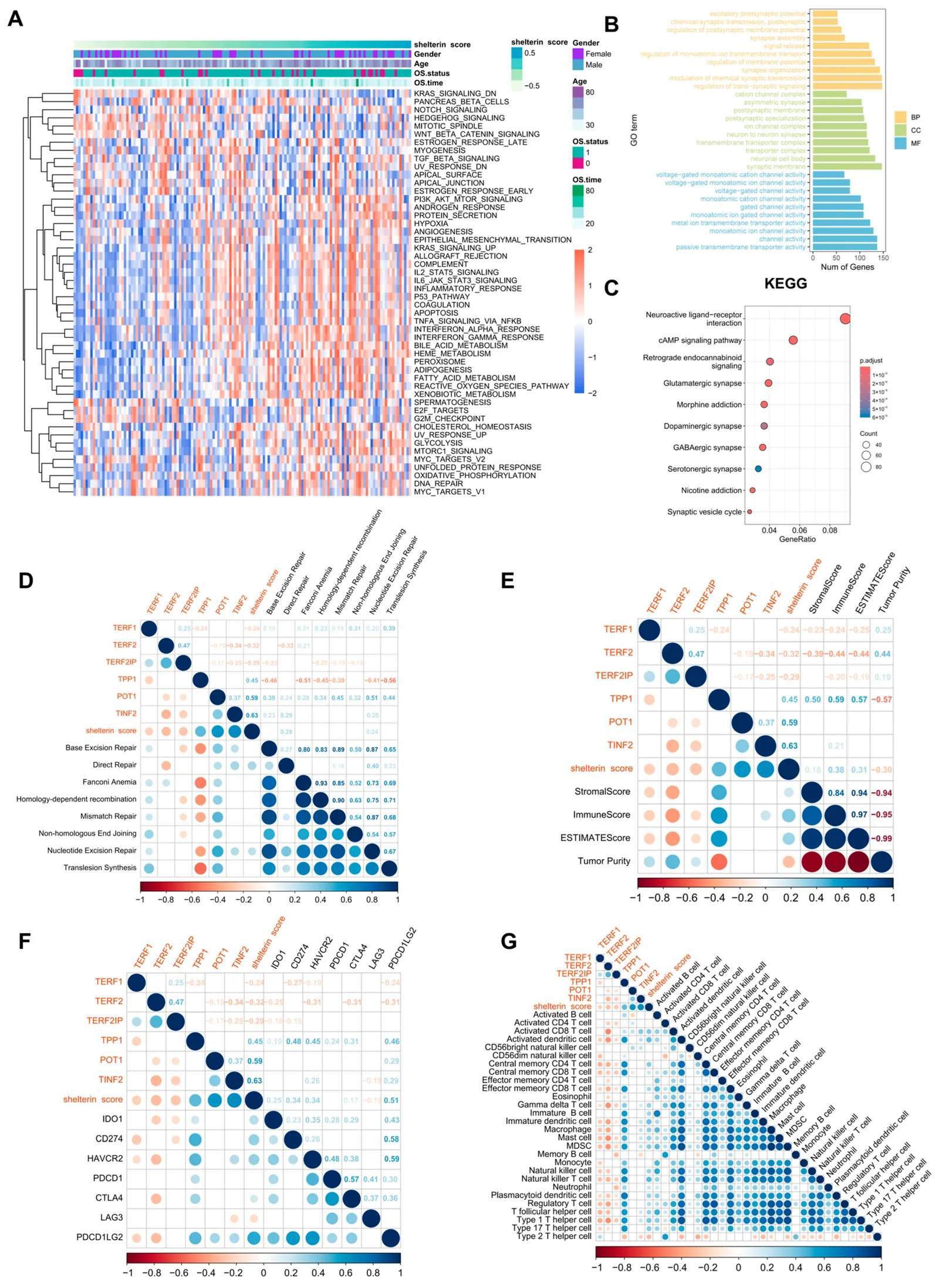
Shelterin score-associated biological pathways, DNA damage repair, tumor microenvironment features, and immune correlates in GBM. (**A**) Heatmap of Hallmark pathway activity scores computed by ssGSEA, with samples ordered by shelterin score. (**B**) Gene Ontology (GO) enrichment analysis of shelterin-correlated genes. (**C**) Kyoto Encyclopedia of Genes and Genomes (KEGG) pathway enrichment of shelterin-correlated genes. (**D**) Spearman correlations between the shelterin score and DNA damage repair (DDR) pathway activity and immune checkpoint gene expression. (**E**) Spearman correlations between the shelterin score and ESTIMATE-derived stromal, immune, and ESTIMATE scores and tumor purity. (**F**) Spearman correlations between the shelterin score and immune cell infiltration levels. (**G**) Spearman correlations between the shelterin score and immune cell subset infiltration levels.

Further analysis of the TCGA-GBM cohort confirmed that the shelterin score was positively correlated with the activity of multiple DDR pathways, including direct repair and nucleotide excision repair (NER) (Figure 2D), indicating that shelterin complex expression is tightly coupled to the DDR capacity of GBM cells. TME analysis showed that the stromal score, immune score, and ESTIMATE score were all positively correlated with the shelterin score, whereas tumor purity was inversely correlated (Figure 2E), indicating that higher shelterin scores were associated with greater stromal and immune infiltration. The shelterin score was also positively correlated with several immune checkpoint genes implicated in immune evasion (Figure 2D), including IDO1, CD274 (PD-L1), HAVCR2 (TIM-3), CTLA4, and PDCD1LG2 (PD-L2), and with infiltration of activated CD8^+^ T cells, central memory CD4^+^ and CD8^+^ T cells, and natural killer (NK) cells. GO analysis of shelterin-correlated genes identified enrichment in synaptic signaling and ion channel regulation; KEGG analysis highlighted neuroactive ligand–receptor interaction and neurotransmitter synapse signaling pathways.

### 3.3. Construction and Validation of a Machine Learning-Based Shelterin-Related Prognostic Signature

**Figure 3:**
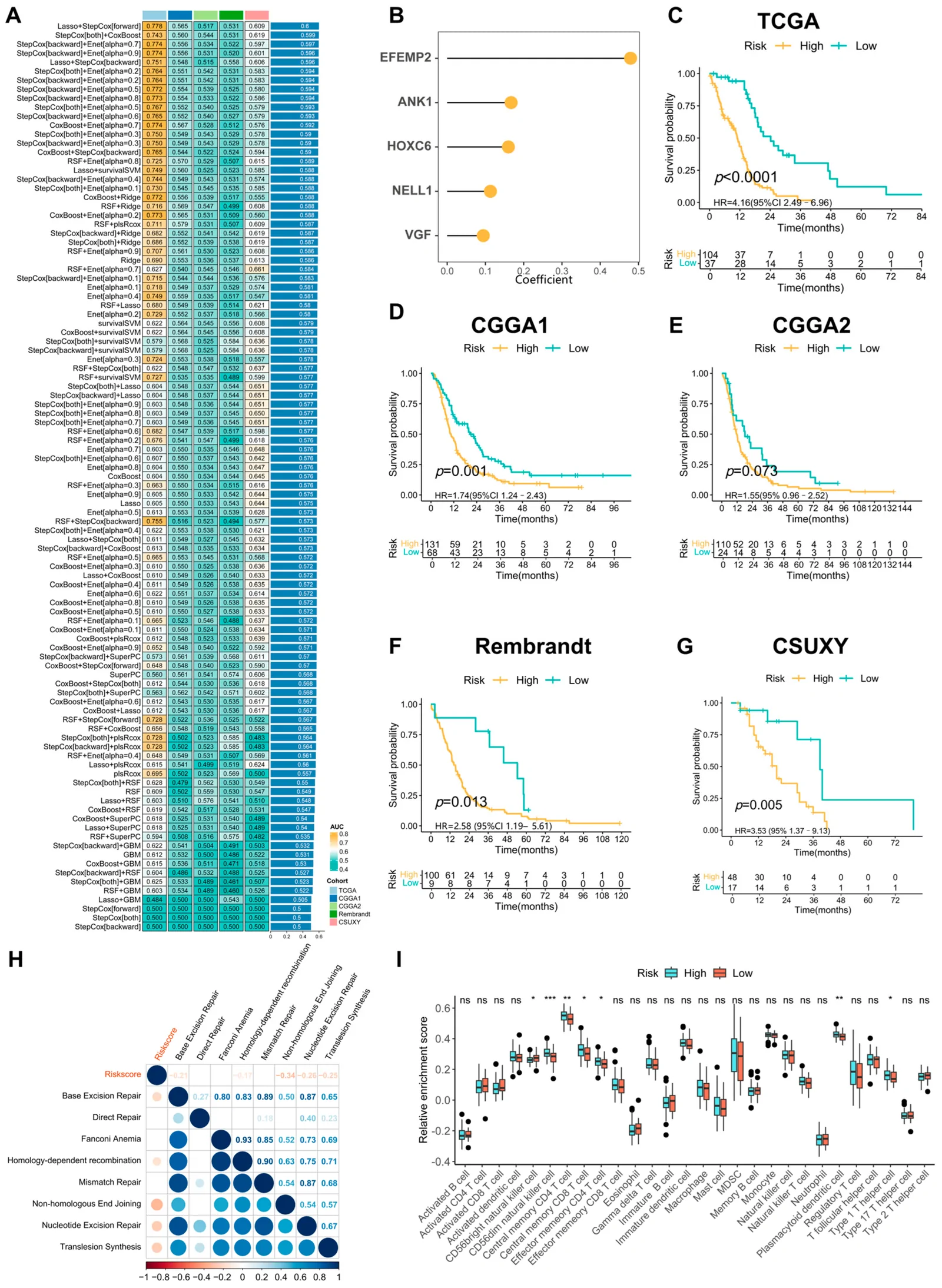
Construction and validation of the machine learning-based shelterin-related prognostic signature (SRS). (**A**) Heatmap of Harrell’s concordance index (C-index) for 101 candidate model configurations generated by combining ten machine learning algorithms; the Lasso + StepCox (forward) combination was selected as optimal. (**B**) Regression coefficients of the top five shelterin-related genes (SRGs) retained in the final multivariable Cox proportional hazards model. (**C**–**G**) Kaplan–Meier overall survival (OS) curves comparing high- and low-risk groups stratified by the SRS risk score in the TCGA, CGGA1, CGGA2, REMBRANDT, and CSUXY cohorts; *p* values were determined by the log-rank test. *p* values were determined by log-rank test and HR by Cox regression. (**H**) Spearman correlations between the SRS risk score and DDR pathway activity. (**I**) Grouped box plots comparing ssGSEA-derived relative enrichment scores of 28 immune cell subtypes between SRS high-risk and low-risk groups. Statistical significance is indicated as * *p* < 0.05, ** *p* < 0.01, and *** *p* < 0.001.

All 101 model combinations were trained on TCGA-GBM and evaluated across cohorts (Figure 3A). The Lasso-StepCox (forward) combination showed the most stable performance, achieving the highest mean C-index of 0.60. This value indicates moderate discrimination rather than clinical-ready predictive accuracy. The model retained five prognostically significant SRGs, regression coefficients were derived from multivariable Cox regression (Figure 3B), and an individualized SRS risk score was computed for each patient. Kaplan–Meier analysis confirmed that high-risk patients had worse OS than low-risk patients in TCGA (HR = 4.16, 95% CI: 2.49–6.96, *p* < 0.0001), CGGA1 (HR = 1.74, 95% CI: 1.24–2.43, *p* = 0.001), REMBRANDT (HR = 2.58, 95% CI: 1.19–5.61, *p* = 0.013), and CSUXY (HR = 3.53, 95% CI: 1.37–9.13, *p* = 0.005), with a non-significant trend in CGGA2 (HR = 1.55, 95% CI: 0.96–2.52, *p* = 0.073) (Figure 3C–G).

The SRS risk score was positively correlated with activity across multiple DDR pathways, including base excision repair, non-homologous end joining (NHEJ), and NER (Figure 3H). Similarly, the grouped box plots showed that the ssGSEA-derived relative enrichment scores of CD56 bright and CD56 dim NK cells, central memory CD4^+^ and CD8^+^ T cells, as well as effector memory CD4^+^ T cells, were significantly elevated in the SRS high-risk group relative to the low-risk group (Figure 3I).

### 3.4. EFEMP2 Is a Core Prognostic Gene Associated with Immune Remodeling, DNA Repair Dysregulation, and Immunotherapy Response

**Figure 4:**
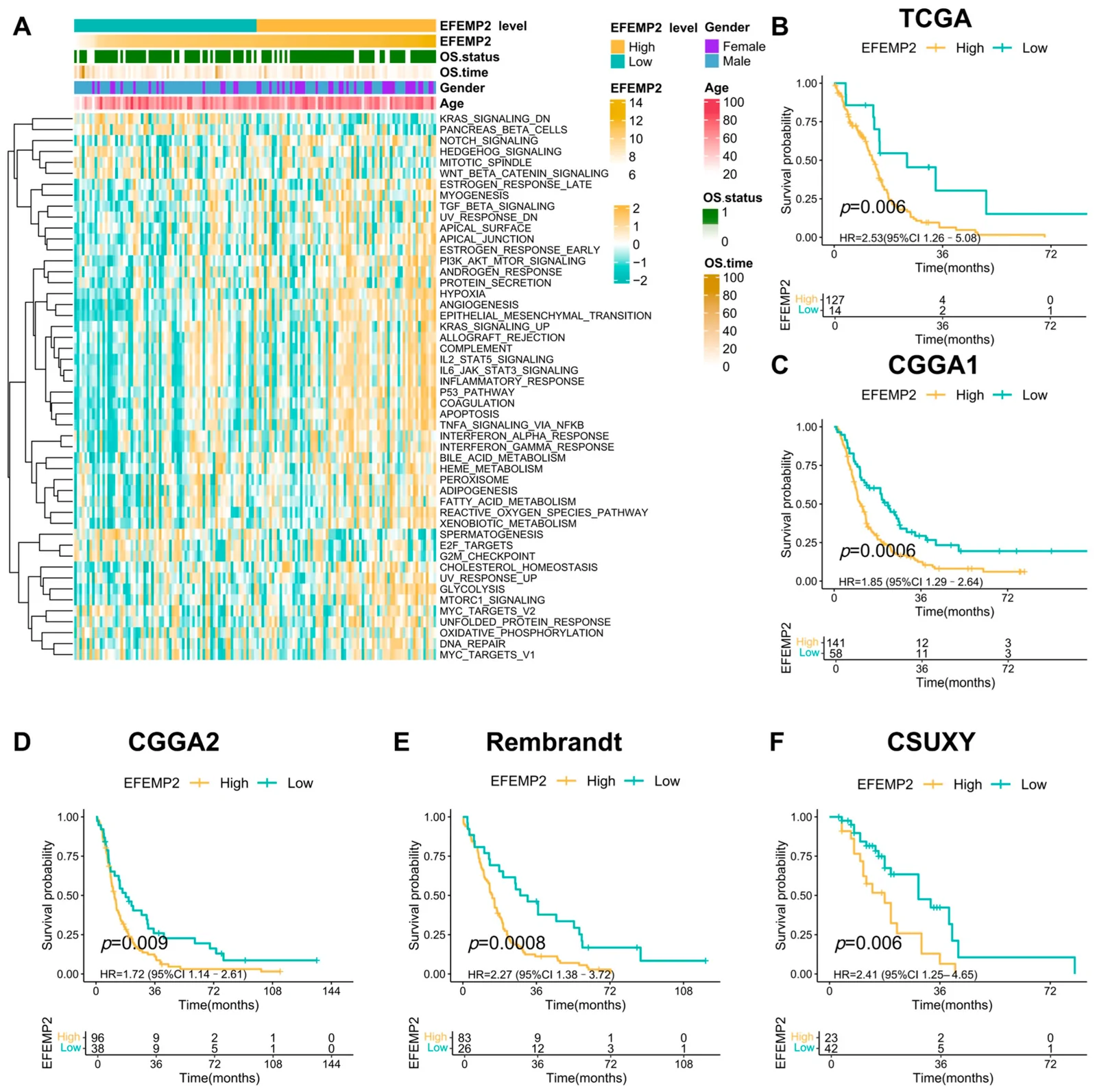
Prognostic and pathway associations of EFEMP2 in GBM. (**A**) Heatmap of Hallmark pathway activity scores with samples ordered by EFEMP2 expression, showing enrichment of cell survival and proliferation pathways in the high-EFEMP2 group. (**B**–**F**) Kaplan–Meier OS curves for high- and low-EFEMP2 expression groups in five independent cohorts (TCGA, CGGA1, CGGA2, REMBRANDT, and CSUXY); *p* values were determined by the log-rank test; HR by Cox regression.

EFEMP2 carried the largest coefficient in the SRS model and was therefore selected for detailed analysis. Kaplan–Meier analysis showed that high EFEMP2 expression was associated with inferior OS in TCGA (HR = 2.53, 95% CI: 1.26–5.08, *p* = 0.006), CGGA1 (HR = 1.85, 95% CI: 1.29–2.64, *p* = 0.0006), CGGA2 (HR = 1.72, 95% CI: 1.14–2.61, *p* = 0.009), REMBRANDT (HR = 2.27, 95% CI: 1.38–3.72, *p* = 0.0008), and CSUXY (HR = 2.41, 95% CI: 1.25–4.65, *p* = 0.006) (Figure 4B–F). ssGSEA revealed that high-EFEMP2 tumors were enriched for pathways related to cell survival, proliferation, and TME adaptation, while low-EFEMP2 tumors showed greater enrichment of cell-cycle signatures (Figure 4A).

**Figure 5:**
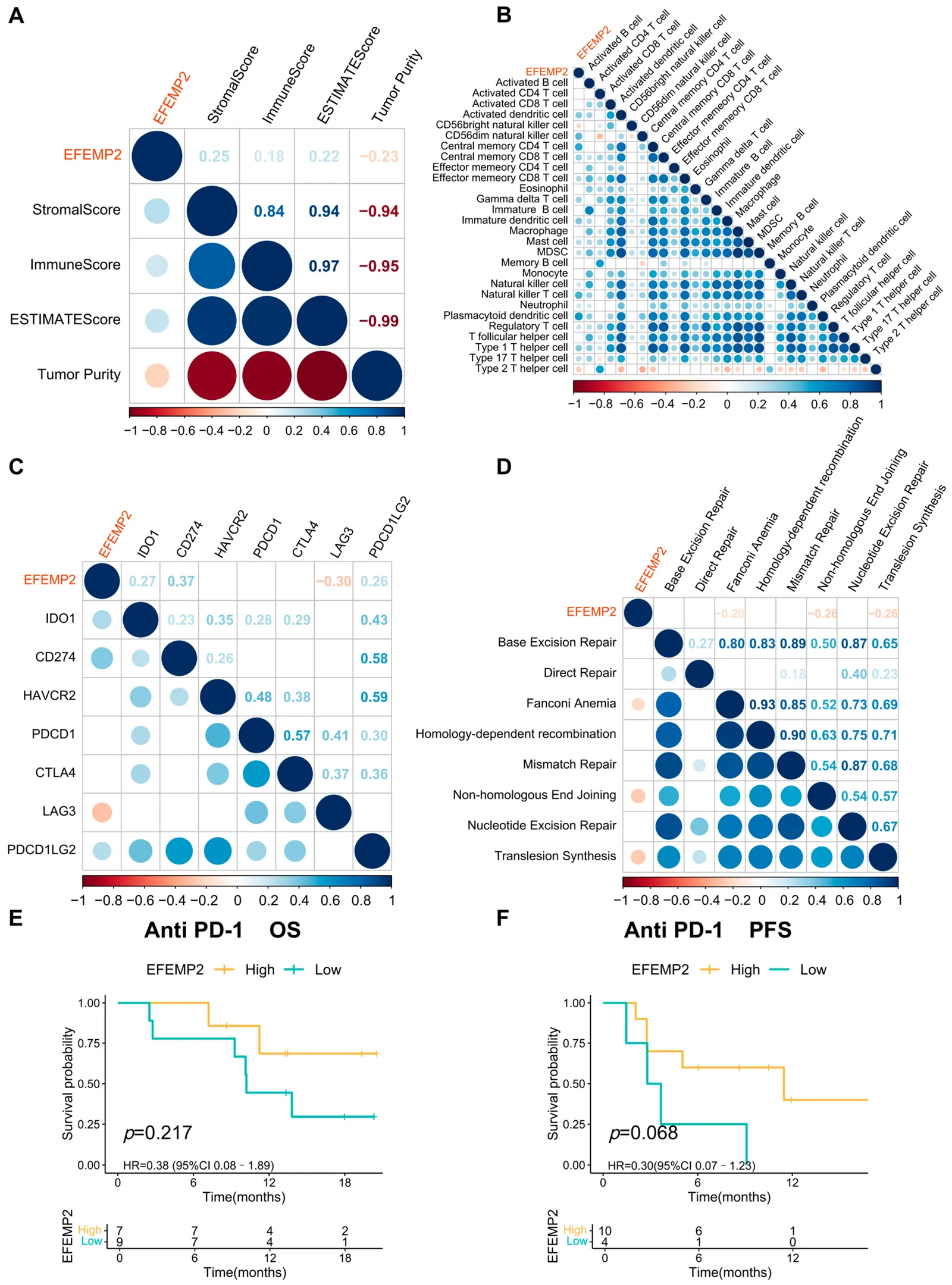
EFEMP2 associations with the tumor microenvironment, immune infiltration, DNA damage repair, and immunotherapy response. (**A**) Spearman correlations between EFEMP2 expression and ESTIMATE-derived stromal, immune, and ESTIMATE scores and tumor purity. (**B**) Spearman correlations between EFEMP2 expression and immune cell subset infiltration levels. (**C**) Spearman correlations between EFEMP2 expression and immune checkpoint gene expression, including IDO1, CD274 (PD-L1), and PDCD1LG2 (PD-L2). (**D**) Gene set enrichment analysis (GSEA) showing negative correlations between EFEMP2 expression and DDR pathways. (**E**,**F**) Kaplan–Meier curves comparing OS and progression-free survival (PFS) between high- and low-EFEMP2 expression groups in the anti-PD-1 immunotherapy cohort; *p* values were determined by the log-rank test; HR by Cox regression. Because neither endpoint reached statistical significance, these findings should be interpreted as exploratory.

Analysis of TME features confirmed that the stromal, immune, and ESTIMATE scores were positively correlated with EFEMP2 expression, whereas tumor purity was inversely correlated (Figure 5A). CIBERSORT-based deconvolution showed that EFEMP2 expression was associated with multiple immune cell populations, particularly central memory CD4^+^ and CD8^+^ T cells, NKT cells, and MDSCs (Figure 5B). Immune checkpoint analysis revealed positive correlations between EFEMP2 and IDO1, CD274 (PD-L1), and PDCD1LG2 (PD-L2) (Figure 5C). Gene set enrichment analysis (GSEA) further demonstrated that EFEMP2 expression was negatively correlated with several DDR pathways, including the Fanconi anemia pathway, non-homologous repair pathways, NHEJ, and translesion synthesis (TLS) (Figure 5D), suggesting that high EFEMP2 expression is accompanied by compromised DNA repair and heightened genomic instability.

In the anti-PD-1 immunotherapy cohort, patients with high EFEMP2 expression showed a non-significant trend toward longer OS (HR = 0.38, 95% CI: 0.08–1.89, *p* = 0.217) and PFS (HR = 0.30, 95% CI: 0.07–1.23, *p* = 0.068) relative to those with low expression (Figure 5E,F). Given the small sample size and lack of statistical significance, these findings should be interpreted as exploratory and hypothesis-generating rather than as evidence that EFEMP2 predicts immunotherapy response.

### 3.5. EFEMP2 Knockdown Suppresses Malignant Phenotypes and Induces DNA Damage in Tumor Cells

**Figure 6:**
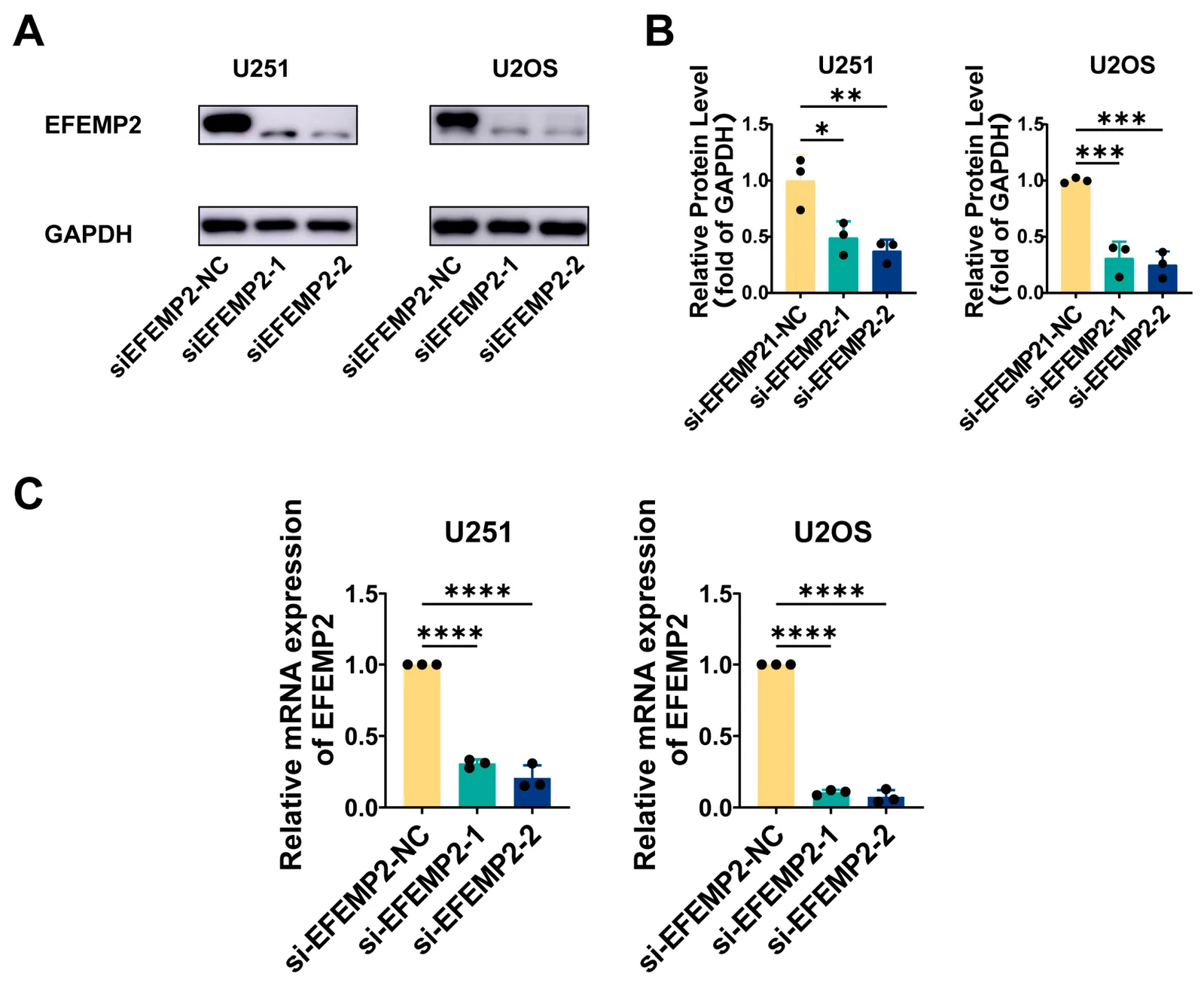
Verification of siRNA-mediated EFEMP2 silencing in U251 and U2OS cells. U251 and U2OS cells were transfected with two independent EFEMP2-targeting siRNAs (siEFEMP2-1 and siEFEMP2-2) or a non-targeting negative control siRNA (siEFEMP2-NC). (**A**,**B**) Western blot analysis of EFEMP2 protein expression in U251 and U2OS cells following siRNA transfection; GAPDH served as the internal loading control. (**C**) Quantification of EFEMP2 mRNA levels by quantitative reverse transcription-polymerase chain reaction (qRT-PCR), normalized to GAPDH. Data are presented as mean ± standard deviation (SD) from three independent experiments. Statistical significance is indicated as * *p* < 0.05, ** *p* < 0.01, *** *p* < 0.001, and **** *p* < 0.0001.

siRNA-mediated knockdown was first validated: both siEFEMP2-1 and siEFEMP2-2 reduced EFEMP2 protein levels in U251 and U2OS cells (Figure 6A,B), and RT-qPCR confirmed corresponding decreases at the mRNA level (Figure 6C).

**Figure 7:**
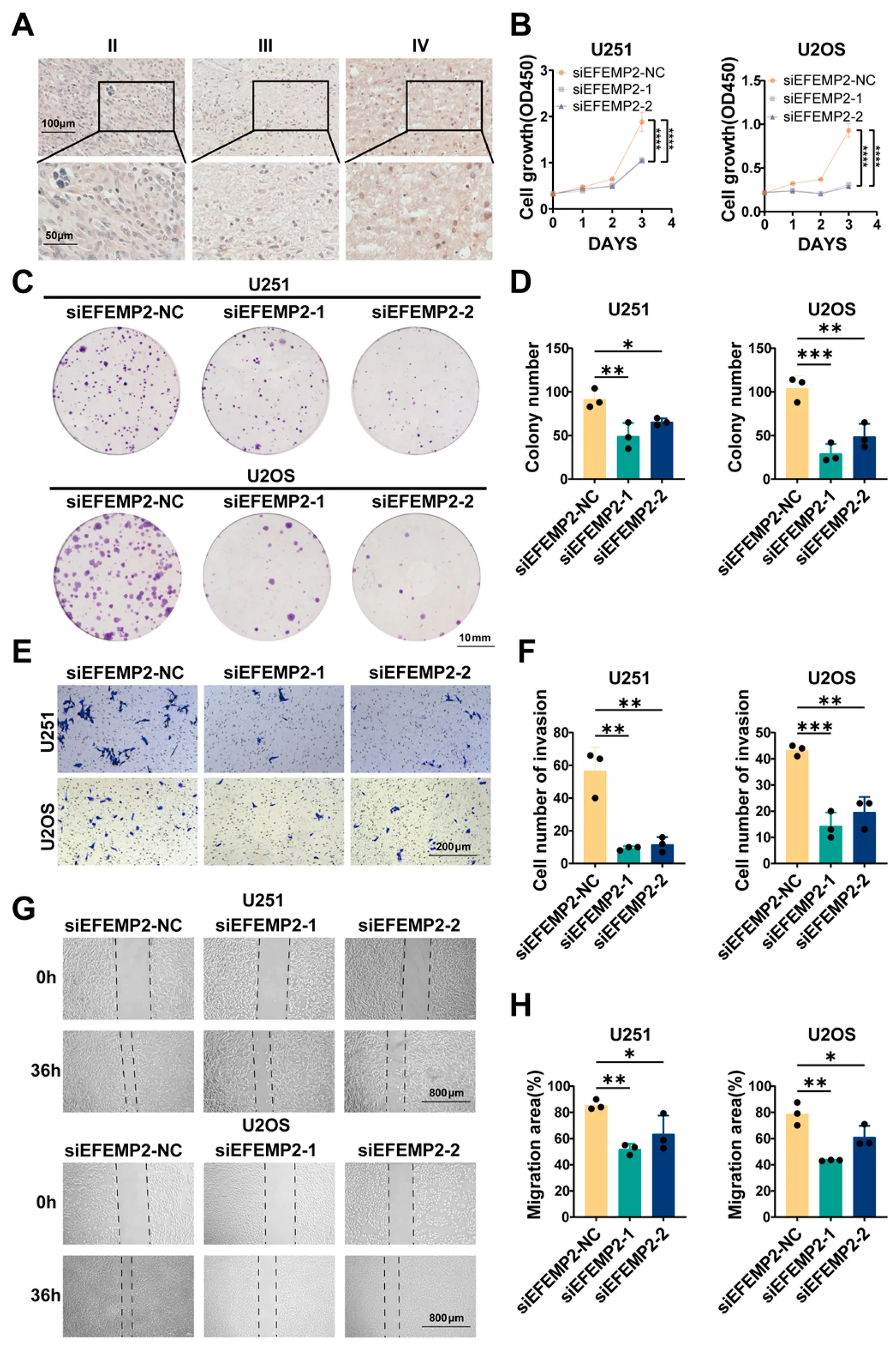
EFEMP2 expression in glioma tissues and its role in regulating tumor cell proliferation, invasion, and migration. (**A**) Representative immunohistochemical (IHC) images showing EFEMP2 protein expression in glioma specimens across different WHO grades; EFEMP2 staining was predominantly cytoplasmic and membranous, with staining intensity and positivity rate increasing with tumor grade. (**B**) Cell Counting Kit-8 (CCK-8) proliferation assay growth curves demonstrating the effects of EFEMP2 silencing on U251 and U2OS cell viability measured at days 0, 1, 2, and 3. (**C**,**D**) Colony formation assays showing changes in clonogenic capacity following EFEMP2 knockdown in U251 and U2OS cells. (**E**,**F**) Transwell invasion assays evaluating the impact of EFEMP2 depletion on the invasive ability of U251 and U2OS cells; cells migrated through Matrigel-coated membranes toward 10% FBS-containing medium. (**G**,**H**) Wound healing assays assessing migratory capacity after EFEMP2 knockdown in U251 and U2OS cells; wound closure was monitored over 36 h. Representative images are presented, and quantitative results are expressed as mean ± SD from three independent experiments. Statistical significance is indicated as * *p* < 0.05, ** *p* < 0.01, *** *p* < 0.001, and **** *p* < 0.0001.

Immunohistochemistry of glioma tissues across WHO grades showed that EFEMP2 protein with predominant cytoplasmic and membranous localization increased in both staining intensity and positivity rate with rising tumor grade, linking EFEMP2 expression to glioma malignancy (Figure 7A).

CCK-8 proliferation assays revealed that EFEMP2-silenced cells grew significantly more slowly than controls from day 1 onward, with the difference widening progressively through day 3 (Figure 7B, *p* < 0.0001). Colony formation assays corroborated these findings: EFEMP2 knockdown markedly reduced colony number in both U251 (siEFEMP2-1, *p* < 0.01; siEFEMP2-2, *p* < 0.05) and U2OS cells (siEFEMP2-1, *p* < 0.001; siEFEMP2-2, *p* < 0.01) (Figure 7C,D).

Transwell and wound-healing assays demonstrated that EFEMP2 silencing significantly attenuated both invasion and migration. In the Transwell assay, the number of invaded cells decreased in U251 (both siRNAs, *p* < 0.01) and U2OS cells (siEFEMP2-1, *p* < 0.001; siEFEMP2-2, *p* < 0.01). Similarly, wound closure was reduced in both cell lines (Figure 7E–H).

**Figure 8:**
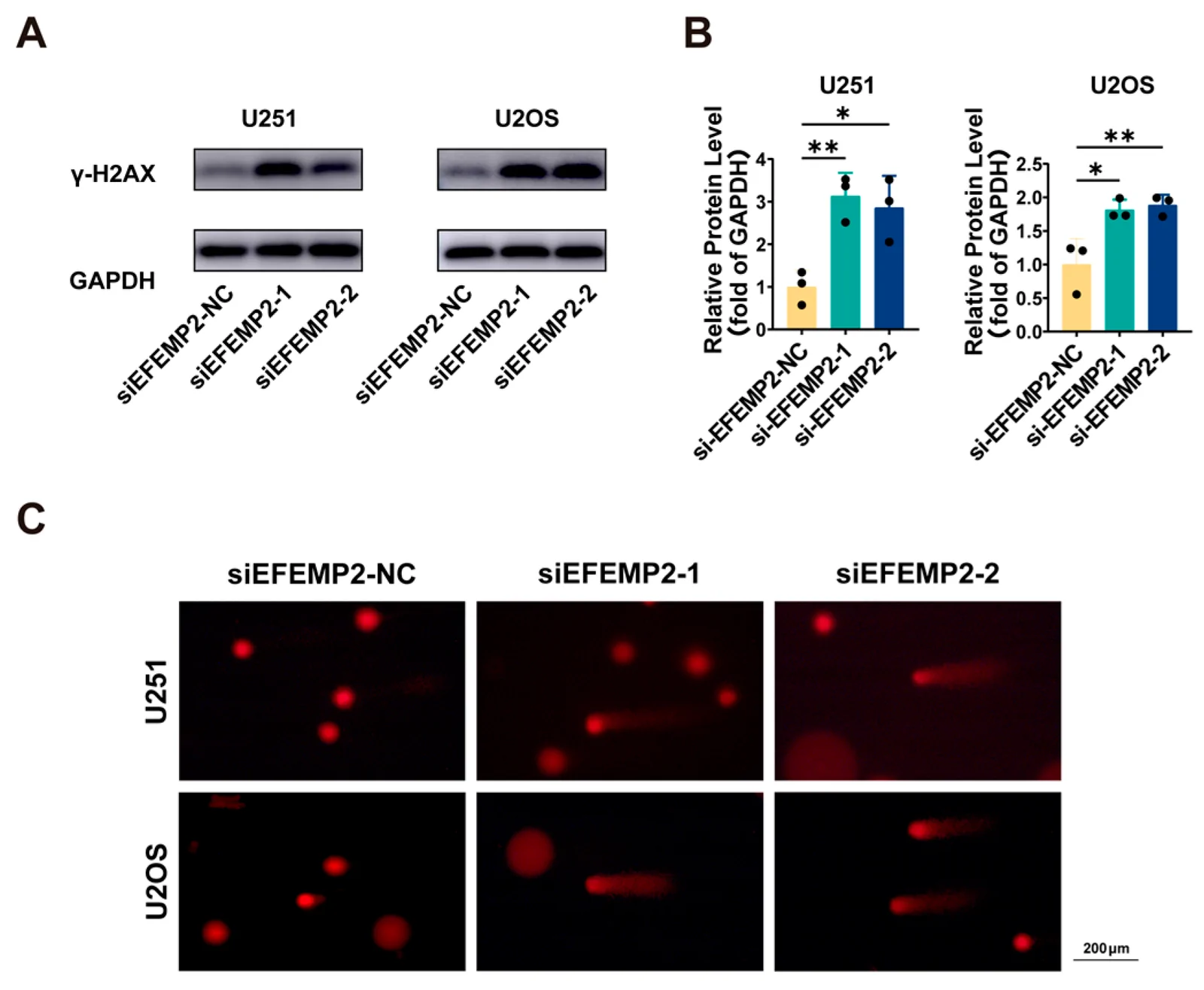
Activation of the DNA damage response following siRNA-mediated depletion of EFEMP2. (**A**,**B**) Western blot analysis of γ-H2AX protein levels in U251 and U2OS cells after EFEMP2 silencing with siEFEMP2-1 and siEFEMP2-2; GAPDH served as the loading control. Densitometric quantification of γ-H2AX band intensity is shown. (**C**) Alkaline comet assay assessing DNA double-strand break (DSB) accumulation in U251 and U2OS cells upon EFEMP2 knockdown; representative fluorescence microscopy images are shown, with increased comet tail length and tail moment indicating enhanced DSB formation. Quantitative results are expressed as mean ± SD from three independent experiments. Statistical significance is indicated as * *p* < 0.05, ** *p* < 0.01.

To assess whether EFEMP2 depletion was accompanied by DNA damage, we examined γ-H2AX and comet assay readouts after EFEMP2 silencing. Western blotting showed that EFEMP2 knockdown elevated γ-H2AX protein levels [35] in U251 (siEFEMP2-1, *p* < 0.01; siEFEMP2-2, *p* < 0.05) and U2OS cells (siEFEMP2-1, *p* < 0.05; siEFEMP2-2, *p* < 0.01) (Figure 8A,B). The comet assay confirmed these results: control cells displayed intact, round nuclei, whereas EFEMP2-silenced cells exhibited prominent comet tails with increased tail length and tail moment (Figure 8C), indicating substantial DSB accumulation upon loss of EFEMP2. These experiments did not include radiation-treatment controls or γ-H2AX immunofluorescence imaging and therefore do not establish radiosensitization.

## 4. Discussion

The present study provides a systematic dissection of shelterin-related molecular patterns in GBM, demonstrating that these telomere-associated signatures are intertwined with DDR capacity, TME remodeling, and clinical prognosis. Through integration of multi-cohort transcriptomic data with machine learning and functional validation, we developed an externally evaluated SRS and identified EFEMP2 as a critical regulator bridging genomic instability and immune modulation.

Both the shelterin score and the SRS risk score exhibited positive correlations with multiple DNA repair programs, including NER, base excision repair, and NHEJ. This observation is consistent with the established role of the shelterin complex in maintaining telomere integrity and suppressing inappropriate DNA damage signaling [6,36]. Enhanced DDR capacity is a well-documented contributor to resistance against radiotherapy and temozolomide in GBM [37,38]. Although not all shelterin subunits demonstrated independent prognostic significance, TPP1 and POT1 did segregate with survival outcomes. This selective association has biological plausibility: the TPP1-POT1 heterodimer directly protects telomeric single-stranded DNA, recruits telomerase, and buffers replication stress [13,39,40], suggesting that GBM cells may rely preferentially on specific telomere-protection modules rather than uniform upregulation of the entire complex [15,41].

Beyond tumor-intrinsic biology, shelterin-related signatures displayed notable associations with the immune microenvironment. Elevated shelterin scores correlated with increased immune and stromal infiltration alongside elevated expression of PD-L1, PD-L2, IDO1, CTLA4, and TIM-3 [42]. This concurrent presence of robust immune cell recruitment and an immunosuppressive phenotype is a hallmark of the GBM microenvironment and reflects the predominance of dysfunctional or exhausted immune populations [43,44]. The concurrent positive correlation between shelterin activity, T-cell and NK-cell infiltration, and immune checkpoint upregulation is consistent with the concept of an “inflamed but suppressed” tumor phenotype [45,46]. The enrichment of MDSCs in association with EFEMP2 expression lends additional support to a model in which shelterin-related networks participate in myeloid-driven immune escape, in line with emerging paradigms of myeloid-mediated immunosuppression in GBM [45,47,48].

The machine learning-derived SRS was externally evaluated across multiple independent cohorts and demonstrated consistent but moderate prognostic performance. Relative to single-gene biomarkers, this integrative signature captures the coordinated activity of SRGs and provides a more nuanced representation of tumor biology [49]. A mean C-index of 0.60 should not be interpreted as sufficient for immediate clinical decision-making. Moreover, because 101 candidate model configurations were evaluated without a fully nested cross-validation framework, model-selection optimism may have inflated the apparent discrimination of the selected model. Rather, the value of the SRS lies in its consistent external evaluation across datasets and biological interpretability, because it links shelterin-related transcriptional features with DDR pathway activity, immune infiltration, and prognosis. In the context of precision oncology, such multidimensional signatures could facilitate patient stratification for personalized regimens that combine DDR-targeting agents with immunotherapy [50,51]. However, GBM survival is strongly influenced by treatment, surgical variables, molecular subtype, and clinical factors that are incompletely annotated across public datasets. Therefore, prospective validation and integration with established clinical covariates will be required before clinical application.

Among the genes retained in the model, EFEMP2 stood out as the dominant contributor to risk stratification. High EFEMP2 expression was uniformly associated with poor OS across all cohorts, supporting its role as an adverse prognostic marker. At the pathway level, EFEMP2 was linked to tumor cell survival, proliferation, and microenvironmental adaptation [50,52,53,54]. The relationship between shelterin biology and EFEMP2-associated DDR signals should be interpreted cautiously. The shelterin score is a composite measure of the six canonical shelterin subunits and was positively associated with several DDR programs, whereas EFEMP2 is not a core shelterin component but a shelterin-related prognostic gene. Thus, the negative association between EFEMP2 expression and several DDR pathways may reflect a distinct adverse state involving extracellular-matrix remodeling, microenvironmental adaptation, and genomic stress rather than a simple linear extension of the aggregate shelterin score. Functional experiments showed that EFEMP2 silencing increased γ-H2AX and comet-tail formation, supporting an association between EFEMP2 depletion and DNA damage, but the causal hierarchy among EFEMP2, shelterin-related programs, and DDR remains to be defined [35,55].

EFEMP2 also appears to be associated with the interface of genomic maintenance and immune regulation. Its expression correlated positively with the infiltration of memory T cells and NKT cells as well as MDSCs and immune checkpoint molecules including PD-L1 and PD-L2. These correlations are consistent with an immunosuppressive milieu, and it may exert such regulatory effects by promoting myeloid cell recruitment and fostering T-cell dysfunction [56,57]. Although the association between high EFEMP2 expression and improved outcomes in the anti-PD-1 cohort did not reach statistical significance, the observed trend raises a hypothesis that EFEMP2-high tumors, by virtue of their more inflamed microenvironment, may show differential sensitivity to ICB [27,58]. This possibility requires confirmation in larger and prospectively annotated immunotherapy cohorts before EFEMP2 can be considered a predictive biomarker [59].

This study has several limitations that should be considered. First, the anti-PD-1 cohort was modestly sized, limiting statistical power and precluding definitive conclusions regarding EFEMP2 as an immunotherapy-response biomarker. Immunotherapy benefit is shaped by treatment line, molecular background, tumor burden, prior therapeutic exposure, and primary versus recurrent disease status, all of which warrant evaluation in larger cohorts [58,60]. Second, although the institutional CSUXY cohort provided survival-based validation and IHC support, some clinical variables requested for comprehensive cohort characterization, including extent of resection and primary versus recurrent status, were not uniformly available in the supplied annotation table. Third, the analyses of public databases were retrospective and subject to sample heterogeneity and incomplete clinical annotation. Fourth, the causal relationships among shelterin, EFEMP2, DDR, and immune regulation remain to be established, given that current evidence is based largely on correlative analyses and limited in vitro experiments. U2OS was used as a supportive DDR-proficient model rather than a GBM-specific model, and future work should validate EFEMP2 function in patient-derived GBM cells stratified by different biomarker status, irradiation models, γ-H2AX foci imaging, single-cell sequencing, spatial transcriptomics, immune co-culture systems, and mechanistic in vivo models.

## 5. Conclusions

Shelterin complex activity is linked to DNA damage repair, immunosuppressive microenvironment remodeling, and prognosis in GBM. Using an integrative machine learning framework, we constructed a biologically interpretable SRS with moderate cross-cohort prognostic performance and identified EFEMP2 as its core adverse prognostic gene. EFEMP2 knockdown impaired malignant phenotypes and induced DNA damage markers in cell models, while its elevated expression showed a trend toward improved response in an anti-PD-1 cohort. These findings support EFEMP2 as a candidate prognostic marker and biological node for further study, offering new perspectives for the precision management of glioblastoma, but additional mechanistic and prospective validation is required before clinical or immunotherapy-predictive application.

## Data Availability

Publicly available GBM datasets (TCGA, CGGA1, CGGA2, REMBRANDT) were downloaded from GlioVis (http://gliovis.bioinfo.cnio.es/, accessed on 15 June 2026) and the Gene Expression Omnibus database (https://www.ncbi.nlm.nih.gov/geo/, accessed on 15 June 2026). The internal RNA-seq cohort (GSA for Human: HRA007149) has been deposited in the Genome Sequence Archive at the National Genomics Data Center, China National Center for Bioinformation, Chinese Academy of Sciences (https://ngdc.cncb.ac.cn/gsa, accessed on 15 June 2026). Further information is available from the corresponding author upon reasonable request.

## Supplementary Materials

The following supporting information can be downloaded at: https://www.mdpi.com/article/10.3390/biomedicines14081785/s1. Table S1: Shelterin-related Genes: differentially expressed shelterin-related genes (SRGs) between high- and low-shelterin-score groups; Table S2: Prognosis-related Shelterin-related Genes: candidate genes significantly associated with overall survival by univariate Cox regression in the TCGA-GBM training cohort. Table S3: Available clinicopathological and molecular-pathological characteristics of the institutional CSUXY dataset. The source table contains 65 glioma cases in the present analysis, but variables not uniformly available, such as extent of resection and primary/recurrent status, should not be claimed as analyzed unless additional data are provided.

## Abbreviations

ANOVA	analysis of variance
ATM/ATR	Ataxia Telangiectasia Mutated/Ataxia Telangiectasia and Rad3-related
BSA	bovine serum albumin
CCK-8	Cell Counting Kit-8
CGGA	Chinese Glioma Genome Atlas
CI	confidence interval
CIBERSORT	Cell-type Identification By Estimating Relative Subsets Of RNA Transcripts
C-index	concordance index
CSUXY	Central South University Xiangya cohort
CTLA4	cytotoxic T-lymphocyte-associated protein 4
DDR	DNA damage repair
DMEM	Dulbecco’s modified Eagle’s medium
DSB	DNA double-strand break
EFEMP2	EGF-containing fibulin extracellular matrix protein 2
Enet	elastic net
ESTIMATE	Estimation of STromal and Immune cells in MAlignant Tumours using Expression data
FBS	fetal bovine serum
GBM	glioblastoma
GEO	Gene Expression Omnibus
GO	Gene Ontology
GSEA	gene set enrichment analysis
GSVA	gene set variation analysis
HR	hazard ratio
HRP	horseradish peroxidase
ICB	immune checkpoint blockade
IDO1	indoleamine 2,3-dioxygenase 1
IHC	immunohistochemistry
KEGG	Kyoto Encyclopedia of Genes and Genomes
LM22	leukocyte gene signature matrix 22
MDSCs	myeloid-derived suppressor cells
MSigDB	Molecular Signatures Database
NER	nucleotide excision repair
NHEJ	non-homologous end joining
NK	natural killer
NKT	natural killer T
OS	overall survival
PD-1	programmed cell death-1
PD-L1	programmed death-ligand 1
PD-L2	programmed death-ligand 2
PFS	progression-free survival
plsRcox	partial least squares regression for Cox
PVDF	polyvinylidene difluoride
REMBRANDT	Repository for Molecular Brain Neoplasia Data
RIPA	radioimmunoprecipitation assay
RSF	random survival forest
RT-qPCR	reverse transcription quantitative polymerase chain reaction
SD	standard deviation
SDS-PAGE	sodium dodecyl sulfate-polyacrylamide gel electrophoresis
si-NC	non-targeting negative control
siRNA	small interfering RNA
SRS	shelterin-related signature
ssGSEA	single-sample gene set enrichment analysis
StepCox	stepwise Cox regression
SuperPC	supervised principal components
survival-SVM	survival support vector machine
TCGA	The Cancer Genome Atlas
TIM-3	T-cell immunoglobulin and mucin-domain containing-3
TME	tumor microenvironment
TLS	translesion synthesis
WHO	World Health Organization
γ-H2AX	phosphorylated H2A histone family member X
